# Efficacy of Cystic Fibrosis Transmembrane Regulator Corrector C17 in Beta-Sarcoglycanopathy—Assessment of Patient’s Primary Myotubes

**DOI:** 10.3390/ijms252413313

**Published:** 2024-12-11

**Authors:** Martina Scano, Alberto Benetollo, Francesco Dalla Barba, Eylem Emek Akyurek, Marcello Carotti, Roberta Sacchetto, Dorianna Sandonà

**Affiliations:** 1 Department of Biomedical Sciences, University of Padova, Via U. Bassi 58/B, 35131 Padova, Italy; martina.scano@unipd.it (M.S.); alberto.benetollo@phd.unipd.it (A.B.); francesco.dallabarba@phd.unipd.it (F.D.B.); marcello.carotti@unipd.it (M.C.); 2 Department of Comparative Biomedicine and Food Science, University of Padova, Agripolis, Legnaro, 35020 Padova, Italy; eylememek.akyurek@unipd.it (E.E.A.); roberta.sacchetto@unipd.it (R.S.)

**Keywords:** LGMD2E/R4, LGMD2D/R3, pathogenic mechanism, missense mutations, small molecules, patient-derived cells, myotubes, ubiquitin–proteasome system, ERAD

## Abstract

Limb–girdle muscular dystrophy type 2E/R4 (LGMD2E/R4) is a rare disease that currently has no cure. It is caused by defects in the *SGCB* gene, mainly missense mutations, which cause the impairment of the sarcoglycan complex, membrane fragility, and progressive muscle degeneration. Here, we studied the fate of some β-sarcoglycan (β-SG) missense mutants, confirming that, like α-SG missense mutants, they are targeted for degradation through the ubiquitin–proteasome system. These data, collected using HEK-293 cells expressing either the I119F- or Y184C mutants of β-SG, were subsequently confirmed in primary myotubes derived from an LGMD2E/R4 patient carrying a homozygous I92T mutation. The knowledge that β-SG with an amino acid substitution shares a pathway of degradation with α-SG mutants, allowed us to explore the pharmacological approach successfully tested in LGMD2D/R3. Several CFTR correctors, particularly corrector C17, preserved β-SG mutants from degradation and promoted localization at the sarcolemma of the entire SG complex. The presence of the complex, despite containing a mutated subunit, improved sarcolemma integrity, as evidenced by the reduced creatine kinase release from myotubes under hypoosmotic stress. These results suggest that β-SG missense mutants undergo proteasomal degradation as α-SG mutants, and that CFTR correctors, particularly C17, may be used as a potential therapeutic option for recovering and stabilizing the SG complex in patients with sarcoglycanopathies.

## 1. Introduction

β-sarcoglycanopathy or LGMD2E/R4 is a rare autosomal recessive disease, belonging to the family of limb–girdle muscular dystrophies. Together with LGMD2D/R3 (α-sarcoglycanopathy), LGMD2C/R5 (γ-sarcoglycanopathy), and LGMD2F/R6 (δ-sarcoglycanopathy), it is responsible for up to a quarter of all LGMDs [1]. The disease affects mainly the proximal musculature of the upper and lower limbs, with cardiac involvement in more than 60% of patients and 70% of the most severely affected subjects [2]. Calf and tongue hypertrophy are also frequently reported, and monitoring respiratory capacities can help to assess LGMD2E/R4 evolution [3,4]. The disease onset occurs in childhood, even though the clinical phenotype is broad, ranging from severe to milder phenotypes. The disease is progressive, very often causing the loss of ambulation during adolescence [2].

LGMD2C-F/R3-6 is caused by bi-allelic mutations of sarcoglycan (SG)-encoding genes, respectively, *SGCA* (α-SG), *SGCB* (β-SG), *SGCG* (γ -SG), and *SGCD* (δ-SG), leading to a strong reduction in or lack of the mutated protein, as well as of the wild-type partners. A strict correlation has been observed between the residual amount of sarcoglycans present at the sarcolemma and the clinical outcome, with the most severe cases being those in which SGs are totally absent [5,6]. Additionally, it is difficult to establish a genotype–phenotype correlation. In addition to the variability from the type of mutation, the genetic background of the patient being analyzed must also be considered; in fact, a different outcome of the same mutation has been reported not only in different subjects but also between siblings of the same family [7,8,9].

SGs are transmembrane, glycosylated proteins localized at the sarcolemma of the skeletal and cardiac muscle. They are organized in a tetramer which assembles in a stepwise process: β- and δ-SG form a core complex to which first γ-SG and eventually α-SG bind [10]. Because of its key role in complex assembly, the absence of β-SG causes the severest forms of LGMD, characterized by cardiac and respiratory involvement [4,11]. The SG complex is, in turn, part of the dystrophin-associated protein complex (DAPC), which collaborate to stabilize and protect the sarcolemma from the mechanical stress produced during muscle contraction [10].

Mutations in any SGs often have deleterious consequences on the SG complex on the whole, causing it to be disrupted and unable to travel to the correct location of the plasma membrane. This results in sarcolemma instability, muscle fiber damage, hyperCKemia, and progressive muscle wasting [1,12,13].

It has been estimated that about 60% of all SG defects are missense mutations [14]. A few years ago, it was reported that missense mutations in the *SGCA* gene result in a folding defective α-SG, recognized by quality control (QC) and degraded by the ER-associated degradation (ERAD) pathway [15,16,17]. Similarly, in Soheili et al. [18] an analogous fate was described as similar to the fate of some β-, γ- and δ-SG missense mutants. In addition, the inhibition of *α*-mannosidase I activity in cellular models expressing mutated SGs allowed the re-localization of proteins at the cell membrane, suggesting the involvement of the ERAD pathway in β-SG degradation too [18].

A therapy model to treat sarcoglycanopathies is not yet available [13,14]; thus, our efforts must be focused toward identifying novel potential therapeutic approaches.

We have recently demonstrated that small molecules, known as CFTR correctors, which are primarily developed to rescue type II mutants of the cystic fibrosis transmembrane regulator (CFTR) which is defective in folding and trafficking functions [19,20], are effective in recovering missense mutants of α-SG as well. The proof of concept for the efficacy of CFTR correctors in LGMD2D/R3 has been established in cellular models (transfected HEK293 and patient-derived myogenic cells) and mouse models [17,21,22,23]. Importantly, in a mouse model exploring the human R98H α-SG missense mutant in the hind limbs of mice, the myopathic phenotype significantly improved at both the histological and molecular levels after treatment with the CFTR corrector C17. Notably, in the treated animals, the muscle force was recovered to the level of the healthy mice, with an absence of side effects [21].

In light of promising results concerning LGMD2D/R3, here we tested the efficacy of CFTR correctors in LGMD2E/R4. First, we confirmed that β-SG carrying a single amino acid substitution (such as I92T, I119F, and Y184C) follows the same degradative route as α-SG missense mutants and is proteasome-dependent. This evidence was provided using both cell models and primary myogenic cells from an LGMD2E/R4 subject.

As expected, corrector treatments, particularly using C17, were effective in rescuing the SG complex in β-sarcoglycanopathy patients’ myotubes. The mutated sarcoglycan increased in content, assembled with the wild-type partners, and moved to the cell membrane, consequently providing a functional improvement. These results deeply suggest that the ”repair strategy” conducted using selected CFTR correctors successfully applied to cystic fibrosis and LGMD2D/R3 could be feasible in LGMD2E/R4 too.

## 2. Results

### 2.1. β-SG Expressed in HEK-293 Cells

To verify that β-SG carrying a single amino acid substitution follows a degradative pathway analogous to the pathway of α-SG missense mutants [16,17], we transiently transfected human embryonic kidney (HEK) 293 cells with mouse cDNA coding for β-SG, either in the wild-type or mutated form. In Supplementary Figure S1, it is reported that the sequence alignment of the human and mouse β-SG proteins shows an identity of 93.7% and a similarity of 97.5%. Through site-directed mutagenesis, we introduced two different amino acid substitutions, namely I119F and Y184C. The first mutation was primarily identified in an Amish population [24] while the second one was initially identified in a Portuguese patient [25]. Both of them are associated with an intermediate–severe phenotype of the disease. In Supplementary Figure S1, it is possible to note that the sites selected to introduce amino acid substitutions are located in conserved regions between human and mouse β-SG proteins. This allows us to expect that mutations in the mouse sequence will have the same consequences as those in the human sequence.

**Figure 1:**
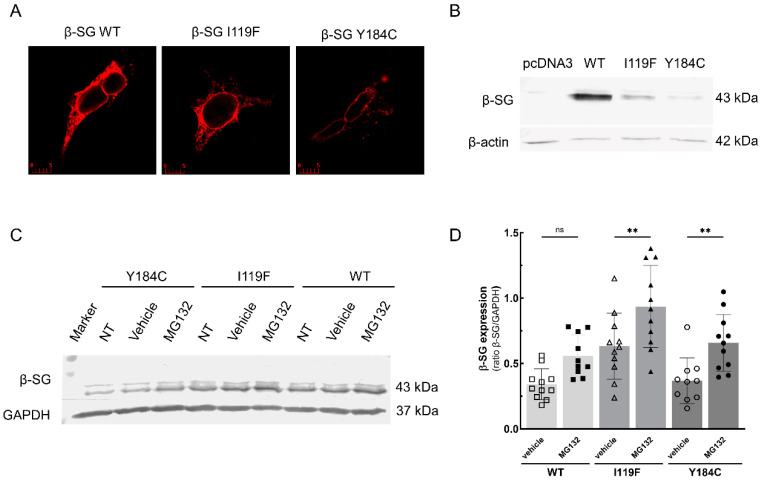
β-SG protein expression in HEK-293 cells. (**A**) Immunofluorescence of HEK-293 cells transiently expressing the wild-type and the mutated forms of β-SG, as indicated. After cell permeabilization, β-SG was recognized by a specific primary rabbit polyclonal antibody and revealed by the Alexa-Fluor488-conjugated anti-rabbit secondary antibody. The images were recorded with a Leica TCS-SP5 confocal microscope using the same setting conditions. Bars indicate 5 µm. (**B**) A representative Western blot (WB) of 20 µg of protein lysates from HEK-293 cell transiently transfected with the pcDNA3 vector carrying the WT or mutated sequence of mouse β-SG; as indicated, pcDNA3 indicates the empty vector. The membrane was probed with primary antibodies to β-SG and β-actin, used as the loading control. (**C**,**D**) A representative WB and densitometric analysis of protein lysates (40 µg) from HEK-293 cells transiently transfected with the expression vector carrying the wild-type and mutated β-SG, as indicated, and either not treated (NT), treated with vehicle (DMSO 1‰), or treated with the proteasome inhibitor MG132 10 µM. The membrane was probed with primary antibodies to β-SG and GAPDH, used as the loading control. Statistical analysis was performed by a one-way ANOVA test followed by Sidak’s multiple comparisons test. The mean values ± SEM are also reported; ns, non-significant; **, *p* ≤ 0.01.

The immunofluorescence analysis of cells expressing the wild-type form of β-SG reported in Figure 1A reveals abundant protein expression, albeit intracellularly confined. This is in accordance with previous evidence that, in the absence of the other SGs, β-SG predominantly localizes intracellularly, around the nucleus [26]. Furthermore, it is known that the membrane localization of SGs relies on the coordinated synthesis and assembly of all four proteins into a molecular complex [26,27,28]. When the mutated forms of β-SG were expressed in the HEK293 cells, it was possible to observe that the proteins were still intracellularly confined, but the overall level was drastically reduced, as evidenced, in particular by the Western blot (WB) analysis of cell protein lysates (a representative WB experiment is reported in Figure 1B). These findings support the idea that the mutated forms of β-SG are recognized by the cell’s quality control system, because of its folding defectivity, and undergo degradation through the ubiquitin–proteasome pathway, like many α-SG mutants [16] and as already suggested by Soheili et al. [18].

### 2.2. Proteasomal Inhibition Reduces the Degradation of β-SG Mutants

To probe the involvement of the ubiquitin–proteasome system in β-SG mutants’ degradation, HEK-293 cells transfected with I119F and Y184C mutants were incubated with either a vehicle (DMSO) or the reversible proteasome inhibitor MG132, a peptide aldehyde that blocks the proteolytic activity of the 26S proteasome complex. Subsequent WB analysis (Figure 1C,D) shows a significant increase in the steady-state level of β-SG mutants after MG132 treatment, further supporting the engagement of the ERAD pathway [15] and, specifically, the proteasomal activity in β-SG mutant degradation. These findings are consistent with the evidence collected for α-SG mutants [16] and confirm what was suggested in [18]. It is interesting to note that, in addition, the wild-type β-SG levels slightly increased upon proteasomal inhibition. Though not statistically significant, this trend suggests that the overexpression itself leads to the accumulation of possibly misfolded proteins that can be degraded by the proteasome and/or that, in the absence of wild-type partners, as in heterologous cells expression, β-SG cannot form the complex, accumulates in the ER and is, at least partially, degraded by the proteasome.

### 2.3. Proteasomal Inhibition Rescues the Sarcoglycan Complex in Primary Myotubes from an LGMD2E/R4 Patient

In support of what was observed with the HEK-293 cells expressing the I114F or Y184C mutants of β-SG, we investigated the efficacy of MG132 in rescuing the SG complex directly in human samples. We had the opportunity to use primary cells derived from a muscular biopsy of an LGMD2E/R4 patient carrying a homozygous c.275 T >C (pI92T) mutation on the *SGCB* gene. Supplementary Figure S1 shows a schematic representation of the β-SG protein topology, showing that this mutation and the two amino acid substitutions studied in the heterologous cell model (I119F and Y184C) are located in the extracellular portion of the protein.

**Figure 2:**
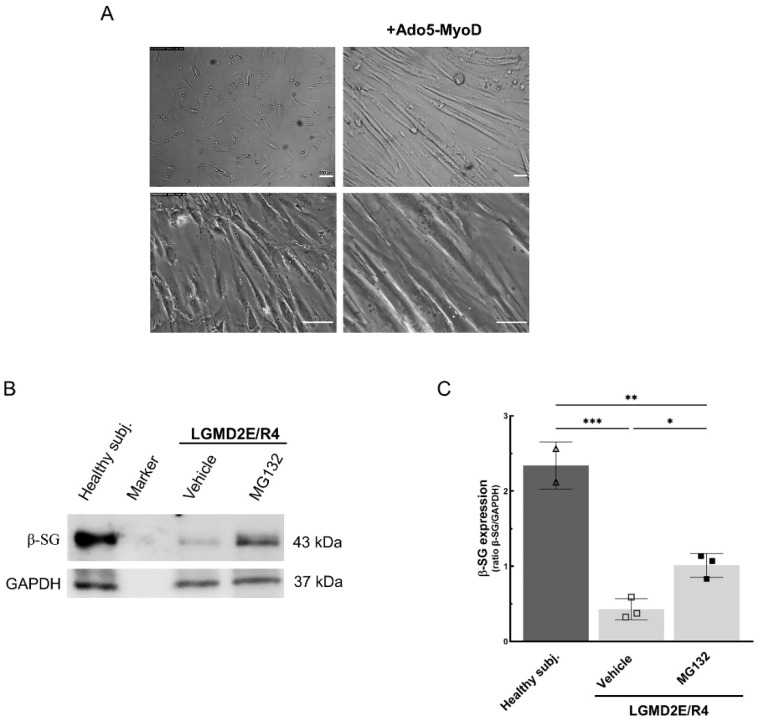
Proteasome inhibition rescued β-SG protein expression in LGMD2E/R4 myotubes carrying the I92T mutation. (**A**) Phase contrast images of the primary cells isolated from the muscular biopsy. To induce differentiation, primary cells were transduced with the Ado5-MyoD virus carrying the master gene of muscle differentiation MYOD (+Ado5-MyoD), and incubated for 7 days in a differentiating medium. Images on the left are the primary cells not transduced with Ado. Bars indicate 20 µm. (**B**,**C**) A representative WB and densitometric analysis of total protein lysates (20 µg) from 7 -day-differentiated myotubes from healthy and LGMD2E/R4 subjects treated for the last 8 h with a vehicle (DMSO 1‰) or MG132 10 µM. Each dot is the average value of one experiment performed in triplicate. The mean value ± SD is also reported. Statistical analysis was performed by a one-way ANOVA test followed by Tukey’s multiple comparisons test; *, *p* ≤ 0.05; **, *p* < 0.01; ***, *p* < 0.001.

Probably due to the advanced state of disease at the time of the biopsy, the resulting cells had a reduced ability to differentiate into myotubes. Therefore, to overcome this issue, the cells were transduced with an adenovirus (Ado5) carrying the sequence coding for the myoblast determination protein 1 (MyoD). This master gene of skeletal muscle development is able to induce the differentiation of unrelated cells into myotubes, multinucleated cells expressing markers of the skeletal muscle lineage [29,30]. Supplementary Figure S2 reports the experiments carried out to determine the concentration (multiplicity of infection MOI) of the Ado5-MyoD virus, which is useful in promoting myotube differentiation from the transduced human fibroblasts. The morphological analysis evidenced the typical elongated tubular shape of the myotubes only in cells where infection occurred. Similarly, the WB analysis highlighted how the MOI of the virus used in transduction determined the level of α-SG, one of the subunits of the SG complex known to be highly expressed in differentiated myotubes [31]. In the subsequent experiments with LGMD2E/R4 cells, we used an MOI of 50, corresponding to a concentration of 35 × 10^6^ PFU (the plaque-forming unit) of the Ado5-MyoD virus. After transduction, LGMD2E/R4 cells started to differentiate, and at 7 days they generated well-differentiated myotubes with the typical elongated shape, as shown in panel A of Figure 2.

Seven-day-old myotubes were treated with the proteasome inhibitor MG132 for the last 8 h to check if the mutated β-SG was indeed degraded through this system. WB analysis showed that the levels of mutated β-SG were significantly increased upon proteasome inhibition. Even though the amount of β-SG was not comparable to the level present in healthy control myotubes, nevertheless, proteasomal inhibition resulted in a 2.5-fold increase in the protein, in comparison to vehicle-treated cells (Figure 2B,C). This strongly suggests the proteasome-dependent degradation of β-SG mutants, as observed in cell models (Figure 1C,D) and previously established for α-SG mutants [15,16].

### 2.4. CFTR Corrector Treatments Rescue the SG Complex in Primary Myotubes from an LGMD2E/R4 Patient

**Figure 3:**
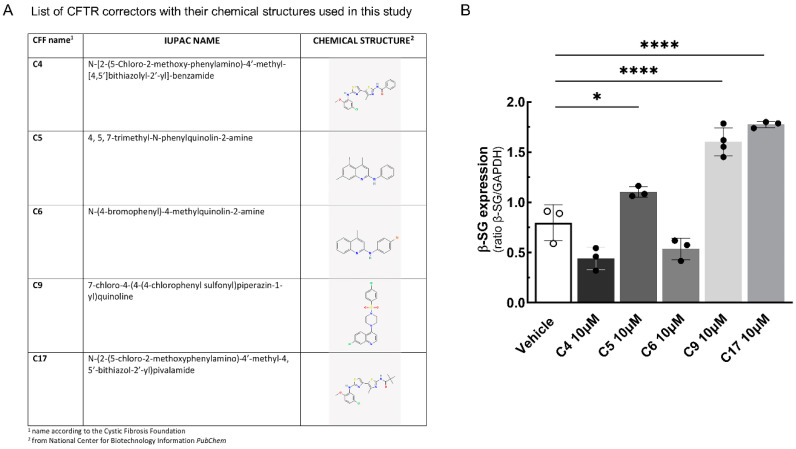
CFTR corrector treatments rescued I92T-β-SG protein expression in LGMD2E/R4 myotubes. (**A**) A table listing the CFTR corrector used in this study. For each CFTR corrector, the name according to the cystic fibrosis foundation, the IUPAC formula, and the chemical structure are reported. (**B**) Densitometric analysis of WB experiments carried out with protein lysates from 7-day-differentiated myotubes from healthy and LGMD2E/R4 subjects treated for the last 48 h with a vehicle (DMSO 1‰) or the indicated CFTR correctors. Each dot is the average value of one experiment performed in triplicate. The mean values ± SD are also reported. Statistical analysis was performed by a one-way ANOVA test followed by Dunnett’s multiple comparisons test; *, *p* ≤ 0.05; ****, *p* < 0.0001.

Our data evidenced that β-SG missense mutants (I119F and Y184C) follow a degradative pathway similar to the one described for α-SG mutants, suggesting that β-SG missense mutants are also folding-defective. If this is the case, the use of the same compounds, CFTR correctors, able to promote the rescue of a defective α-SG, may also be of benefit for β-SG mutants [22,23]. Some CFTR correctors, like those approved for the treatment of the ΔF508 mutation in cystic fibrosis (VX661, VX445, and VX809), exert their action as true pharmacological chaperones, which bind and stabilize the mutated CFTR. Others are primarily modulators of cell proteostasis and are thought to indirectly promote the folding/stabilization of mutants; thus, they are assumed to have broader activity [32,33]. Therefore, the LGMD2E/R4 myotubes were treated with CFTR correctors like C4, C5, C6, C9, and C17, effective in recovering not only the ΔF508-CFTR but also α-SG mutants [23]. The chemical formulas of these correctors are listed in the table of Figure 3A. Figure 3B shows that after 48 h of incubation, the correctors C5, C9, and C17, at a 10 µM concentration, induced a statistically significant increase in β-SG protein content. On the contrary, 48 h of incubation with 10 μM C4 and C6 were ineffective.

**Figure 4:**
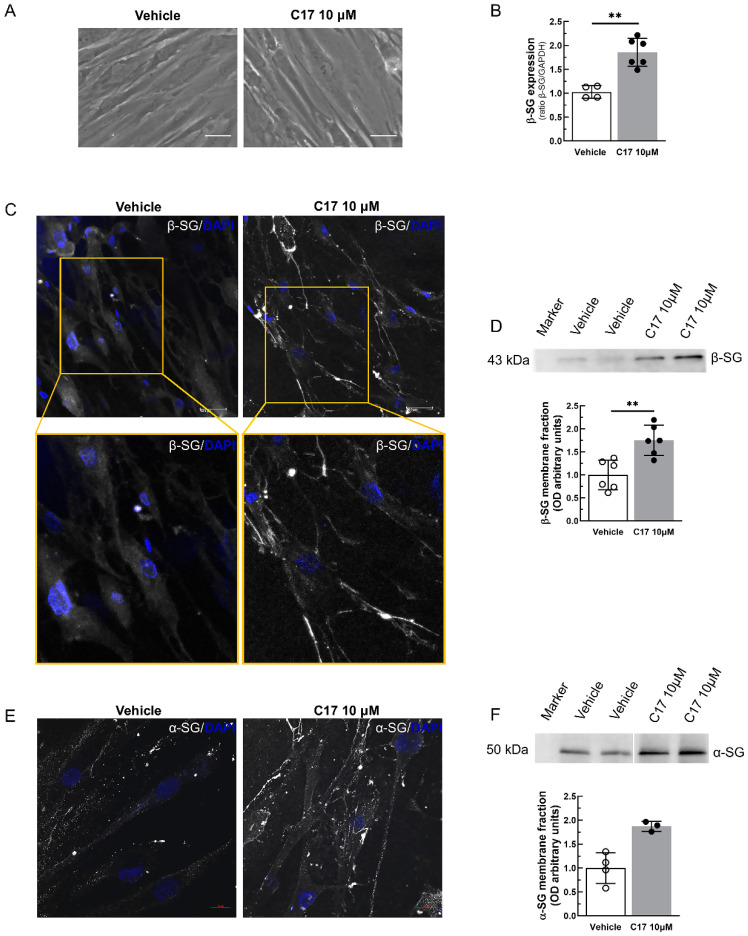
Incubation with CFTR corrector C17 rescued the SG complex at the sarcolemma of LGMD2E/R4 myotubes expressing I92T-β-SG. (**A**) Phase contrast images of MyoD-transduced LGMD2E/R4 myotubes after 7 days of differentiation, treated for the last 96 h with either a vehicle (1‰ DMSO) or C17 10 µM. No signs of toxicity are evident. (**B**) Densitometric analysis of WB of the total protein lysates from 7day-differentiated myotubes treated with either a vehicle (1‰ DMSO) or C17 10 µM. (**C**,**E**) Immunofluorescence images of MyoD-transduced LGMD2E/R4 myotubes after 7 days of differentiation treated for the last 72 h with either a vehicle (1‰ DMSO) or C17 10 µM. Myotubes were labeled with rabbit polyclonal antibodies specific to β-SG (**C**), or α-SG (E) in both cases, recognizing an extracellular epitope of the two proteins. The primary antibodies were revealed by the secondary Alexa-Fluor 488-conjugated anti-rabbit antibody (white signal), and nuclei were revealed by DAPI (blue signal). Bars indicate 50 µm in (**C**) and 20 µm in (**E**). Images were recorded with a Leica TCS-SP5 (**C**) or with a Zeiss LSM 800 (**E**) confocal laser scanning microscopes with the same setting conditions. (**D**,**F**) Representative WBs and densitometric analysis of plasma membrane resident proteins isolated by biotinylation and streptavidin-chromatography, from 7day-differentiated myotubes treated for the last 72 h with either a vehicle (1‰ DMSO) or C17 10 µM. The membranes were probed with antibodies specific to β-SG (**D**) or α-SG (**F**). Each dot is the average value of one experiment performed at least in duplicate. The mean value ± SD is also reported. Statistical analysis was performed by a Mann–Whitney test; ** *p* ≤ 0.01.

Ado5-MyoD-transduced myotubes were then incubated with the most effective corrector, C17, for 96 h. Figure 4B shows that 10 μM of C17 induced a two-fold increase in the β-SG content in comparison to the vehicle-treated myotubes, with no major alterations in the morphology of the elongated myotubes and no signs of cytotoxicity, such as membrane blebbing or cells detaching from the plate (Figure 4A). This suggests that the treatment was safe and successfully recovered the mutated β-SG in the pathological samples. As a further control, we verified the level of differentiation of the Ado5-MyoD-transduced myogenic cells used in the experiments, measuring the expression of the myosin heavy chain (MHC). As is possible to observe in Supplementary Figure S3, similar levels of the marker of differentiation were present in treated and untreated myotubes.

As known, the accumulation of the β-SG mutant is therapeutically effective only if it can localize at the sarcolemma. Therefore, we examined the correct localization of β-SG at the cell surface of the LGMD2E/R4 myotubes after 72 h of treatment with either 1‰ DMSO (vehicle) or 10 μM of C17. Figure 4C reports the immunofluorescence staining of 7day-old myotubes decorated with a primary antibody specifically recognizing an extracellular epitope of β-SG. Cells treated with the vehicle present a very faint signal, mostly located intracellularly; on the contrary, a clear signal is evident at the cell surface of myotubes treated with corrector C17. To have a quantitative evaluation of the recovery, we performed WB and densitometric analyses of the sarcolemma proteins purified from the myotubes by biotinylation (Figure 4D,E). We checked β- and α-SG, which showed that in comparison to the control, there was a significant (two-fold) increase in both members of the SG complex at the cell surface after the incubation with the small molecule. Of note, in the assembly process of the tetramer, β-SG and δ-SG play a pivotal role, forming a core complex to which γ- and α-SG associate later, moving eventually toward the plasma membrane [28,34]. Thus, the increased amount of the α-SG at the cell surface is strong evidence of the successful recovery of the whole complex at the sarcolemma.

### 2.5. C17 Corrector Treatment Restores Membrane Functionality

The SG complex, crucial for sarcolemma protection during muscle contraction cycles, interacts with dystrophin and dystroglycans. Its absence weakens the sarcolemma, rendering it more susceptible to contraction-induced injury and negatively impacting cellular mechanotransduction [35]. In sarcoglycanopathy, characterized by a severe reduction in or absence of the SG complex from the sarcolemma, one of the initial clinical signs is the elevated serum content of the cytosolic muscle protein creatine kinase (CK) [1,36,37,38].

**Figure 5:**
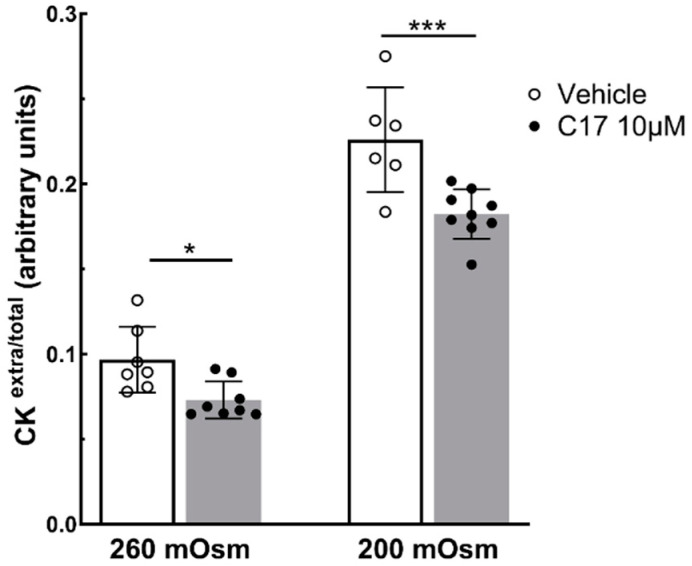
C17 treatment restores membrane functionality to LGMD2E/R4 patients’ myotubes in vitro. Ado5-MyoD-transduced myotubes expressing I92T-β-SG were grown and differentiated for 7 days and treated for the last 72 h with a vehicle (DMSO 1‰) or C17 10 µM. At the end of the treatment, the myotubes were incubated for 20 min in hypo-osmotic solutions, as indicated. Then, the cytosolic protein creatine kinase (CK) was measured in the supernatant of the myotubes, whereas the intracellular level of the protein was determined after cell lysis. Data are plotted as ratios between extracellular and total CK values; the mean values ± SD are also reported. Statistical analysis was performed using a one-way ANOVA test followed by Sidak’s multiple comparisons test; *, *p* ≤ 0.05; *** *p* < 0.001.

Therefore, according to the methodology of [23,39], we measured the CK released by LGMD2E/R4 myotubes in the supernatant after the application of hypotonic conditions to assess whether the rescue of the SG complex by C17 treatment reduced sarcolemma fragility. Seven-day-old myotubes, treated for 72 h with a vehicle or 10 µM of C17, were incubated for 20 min in hypoosmotic conditions (260 and 200 mOsm), and CK release was measured in the supernatant. Figure 5 shows that myotubes treated with the corrector C17 release a statistically significant lower amount of CK in comparison to the vehicle-treated LGMD2E/R4 myotubes. This reduction occurred at both hypoosmotic conditions and was more pronounced at the more stressful condition (200 mOsm). This suggests that, upon corrector C17 treatment, the restored complex assured the functional improvement of the sarcolemma, which became less susceptible to hypoosmotic stress, in comparison to the untreated samples.

## 3. Discussion

LGMD2E/R4 or β-sarcoglycanopathy is a rare autosomal recessive muscular dystrophy, characterized by the progressive degeneration of skeletal muscle tissue and an increase in serum CK due to the fragility of the sarcolemma. Loss of ambulation is a typical occurrence in adolescence, and respiratory problems and cardiac dysfunction as rhythm abnormalities and dilated cardiomyopathy are common complications [2,3]. Only supportive treatments are currently available for sarcoglycanopathies, aiming at ameliorating the quality of life of patients [13,14]. Gene therapy-based approaches for sarcoglycanopathies are currently at an advanced stage of development, with a phase I-II clinical trial for LGMD2E/R4 presently active (NCT03652259) and one for LGMD2C/R5 planned to start soon (NCT05973630). Conversely, no causative therapeutic approach for sarcoglycanopathies based on either stem cells or small molecules have reached clinical trials yet [14,40,41,42]. Thus, any effort devoted to finding a cure for sarcoglycanopathies must be pursued. Consequently, the aim of the present study was to decipher the pathological mechanism of LGMD2E/R4 in vitro and to validate the repurposing of the pharmacological approach based on CFTR correctors, recently validated in vivo for LGMD2D/R3 [17,21,22,23].

SGs are transmembrane glycoproteins that form a tetrameric complex at the sarcolemma of striated muscle. They are co-translationally translocated into the endoplasmic reticulum (ER) for folding, maturation, and assembly into the complex, which eventually enters the secretory pathway to reach the cell surface. In the last few years, it has been shown that single amino acid substitutions in α-SG result in possibly functional but folding-defective proteins, which are recognized by the quality control (QC) machinery of the cell and rapidly degrade through endoplasmic reticulum-associated degradation (ERAD) [15,16,17]. This suggests that the QC system allows for the maturation and correct localization of only native SGs to avoid the accumulation of potentially harmful defective proteins. However, while considerable evidence has been gathered regarding the fate of α-SG mutants [15,16,17], information related to β-SG mutants is still scarce. A recent work by Li et al. [43] studied the outcome of *SGCB* missense mutations, predicting their impact on the formation of the SG complex, but did not evaluate the effects of these variants on the expression levels of the β-SG protein [43]. On the other hand, only the work of Soheili et al. [18] has highlighted how mutants of any sarcoglycan can be substrates of ERAD. In that work, data were collected expressing the mutated β-SG sequences in a heterologous cell model, and the recovery upon treatment was observed, which inhibited the first step of the ERAD pathway [18]. Our present work confirms this observation, showing that two pathogenic mutants (I119F and Y184C) of β-SG, expressed in HEK-293 cells as well as the mutant I92T carried by the myogenic cells from an LGMD2E/R4 subject, are indeed substrates of the executor of ERAD, i.e., the ubiquitin–proteasome system. In fact, the inhibition of the proteasome resulted in a two-fold increase in the content of the β-SG mutants. Unfortunately, the primary cells from this patient had a low differentiative capacity, an issue that was overcome by the viral delivery of *MYOD*, the master gene of skeletal muscle differentiation [44,45,46]. The Ado5-MyoD-transduced cells were thus able to easily differentiate into myotubes maintaining the LGMD2E/R4 signature, with a low expression of sarcoglycans at both the global and sarcolemma level. On the other hand, when these myotubes were treated for just 8 h with the proteasomal inhibitor MG132, the amount of I92T-β-SG had more than doubled, confirming that β-SG mutants are recognized as defective by the QC system of the myotubes, becoming substrates of the ERAD pathway. The knowledge that β-SG missense mutants may share the same fate as α-SG missense mutants [16,17] was of utmost importance when planning the repurposing of the pharmacological approach successfully tested for LGMD2D/R3 [14]. We recently showed that by using small molecules able to “repair” the folding defective α-SG, it is possible to recover a functional SG complex at the sarcolemma. These small molecules belong to the family of CFTR modulators. In particular, we demonstrated that some of the CFTR correctors screened to recover type II mutations of the chloride channel [19] are also effective in inducing the localization of α-SG mutants at the sarcolemma with the reconstitution of the SG complex [22,23]. Based on these results, we tested the same CFTR correctors in Ado5-MyoD-transduced LGMD2E/R4 myotubes, observing that some of them were able to increase the amount of the I92T-β-SG mutant upon an incubation of 48 h. Then, we focused on the corrector C17, the corrector selected as our lead compound, and successfully tested it in vivo in a mouse model of LGMD2D/R3 [21]. As expected, the corrector C17 was able to induce a two-fold increase in the total β-SG after 96 h of incubation of the Ado5-MyoD-transduced LGMD2E/R4 myotubes in the absence of any signs of toxicity. Furthermore, we observed that 72 h of incubation were sufficient to induce the re-localization of the mutated β-SG and the wild-type partner α-SG at the sarcolemma. The evidence that α-SG also trafficked at the sarcolemma after C17 treatment was the proof that the SG complex was correctly rescued. In fact, α-SG is the last subunit that associates with the complex during assembly, and it can be quickly recycled from the membrane in the absence of the other members of the complex [10,28,47]. Notably, the recovered SG complex, despite containing a mutated β-SG, was functional. In fact, when the myotubes were subjected to an in vitro stress test [23,39], a lower amount of the cytosolic CK was released from C17-treated cells in comparison to the vehicle-treated ones. In sarcoglycanopathy, SG-complex disruption leads to sarcolemma fragility and the leakage of CK [1,36,37] as a consequence of the mechanical stress due to muscle contraction. Thus, the reduction in CK leakage from C17-treated myotubes is diagnostic of an improvement of membrane fragility and strongly suggests that the corrector C17 may ameliorate the dystrophic phenotype of the disease.

In conclusion, our data are indicative that CFTR correctors, in particular C17, may have a broad application and should become a valid therapeutic option for sarcoglycanopathy. Still open questions of this strategy are the mechanism of action of CFTR correctors in sarcoglycanopathy, the evaluation of possible side effects, and the broadness of efficacy in a single subtype and all four subtypes of sarcoglycanopathy. Even though not all sarcoglycan missense mutants are expected to be recovered by this strategy, nevertheless, if we consider that these genetic defects are responsible for about the 60% of all sarcoglycan mutations [14], it is possible to infer that a large cohort of patients may benefit of such strategy.

## 4. Materials and Methods

### 4.1. Plasmids, Cell Culture, Transfection, Transduction, and Treatments

Full-length mouse β-sarcoglycan cDNA was generously provided by the RIKEN Genomic Research Group. The sarcoglycan cDNA was cloned in a pcDNA3 expression vector. Point mutations in β-sarcoglycan were engineered using a QuikChange site-directed mutagenesis kit according to the manufacturer’s protocol (Stratagene, La Jolla, CA, USA) with the primer GGCGTCTTCCACCCGCTTTAT for the I119F mutation and GCACGGACTGTGAGACGCAC for Y184C. All constructs were verified through sequencing.

HEK-293 cells were grown in Dulbecco’s modified Eagle’s medium (Thermo Fisher Scientific, Waltham, MA, USA) supplemented with 10% fetal bovine serum (FBS) (Gibco, Thermo Fisher Scientific, Waltham, MA, USA) in a humidified atmosphere containing 5% CO_2_ at 37 °C. For transient expression, HEK 293 cells were seeded at 50,000 cells/well in a 24-well plate and transfected the day after with either WT or a mutated β-SG-expressing vector and Lipofectamine^TM^ 2000 (Thermo Fisher Scientific, Waltham, MA, USA), according to manufacturer’s instructions. MG132 was added 8 h before cell lysis. At the end of the treatments, cells were washed twice with ice cold PBS and lysed with RIPA buffer supplemented with complete protease inhibitors (Sigma-Aldrich, St. Louis, MO, USA).

Primary human myogenic cells from an LGMD2E/R4 patient were isolated from a bioptic fragment from the Telethon Genetic Bio-Bank facility. They were grown in Dulbecco’s modified Eagle’s medium (DMEM) supplemented with 20% FBS (Gibco, Thermo Fisher Scientific, Waltham, MA, USA), insulin 10 µg/mL, Fibroblast Growth Factor (FGF, 25 ng/µL) and Epidermal Growth Factor (EGF, 10 ng/µL). Because they did not spontaneously differentiate, this process was induced by transducing cells with an adenovirus carrying the MyoD ORF (Ado5-MyoD) (Vigene Bioscience, Charles River, Rockville, USA). To define the Ado5-MyoD multiplicity of infection (MOI) able to induce cell differentiation into myotubes, control human fibroblasts were transduced with a different MOI, hence 10, 50, or 100, corresponding to a 7 × 10^6^, 35 × 10^6^, 70 × 10^6^ plaque-forming unit (PFU) in DMEM supplemented with 2% Horse Serum (Euroclone), 10 μg/mL of human recombinant insulin (Sigma-Aldrich), and 100 µg/mL of human Apotransferrin (Sigma-Aldrich, St. Louis, MO, USA). Differentiation was carried out for seven days. At the end of the differentiation period, the cells were washed twice with ice-cold PBS, lysed with RIPA buffer supplemented with a complete protease inhibitor (Sigma-Aldrich, St. Louis, MO, USA) and analyzed by Western blot analysis. The appropriate MOI to transduce LGMD2E/R4 myogenic cells was chosen on the basis of the α-SG expression, known to be highly expressed in differentiated myotubes [31]. Thus, to induce differentiation, LGMD2E/R4 myoblasts, grown at confluence, were incubated with DMEM supplemented with 2% Horse Serum (Euroclone), 10 μg/mL of human recombinant insulin (Sigma-Aldrich), 100 µg/mL of human Apotransferrin (Sigma-Aldrich, St. Louis, MO, USA), and adenovirus carrying the MyoD ORF MOI 50. (Vigene Bioscience, Charles River, Rockville, MD, USA). MG132 was added 8 h before cell lysis, while CFTR correctors were added 48, 72, or 96 h before lysis, according to the specific experiment. After treatments, cells were washed twice with ice cold PBS and lysed with RIPA buffer supplemented with complete protease inhibitor (Sigma-Aldrich, St. Louis, MO, USA).

### 4.2. Chemicals and Antibodies

CFTR correctors C4, C5, C6, C9, and C17 were from Exclusive Chemistry (Kiryat Motzkin, Israel), while proteasome inhibitor MG132 was from Sigma-Aldrich (St. Louis, MO, USA). All compounds were dissolved in DMSO (Sigma-Aldrich, St. Louis, MO, USA) at a 1000× concentration to have the same content of vehicle (1‰) after the dilution at the final concentration used for treatments.

The mouse monoclonal anti β-SG antibody was purchased from Leica Biosystems (Wetzlar, Germany). The rabbit polyclonal antibody specific for α-SG, recognizing the extracellular epitope of the protein, was produced and characterized as previously described [17]. The rabbit polyclonal anti α-SG (ab189254) and anti β-SG (ab83699) were from Abcam (Cambridge, UK); the rabbit polyclonal anti GAPDH (G9545) and mouse monoclonal anti β-actin (A5441) were from Sigma-Aldrich (St. Louis, MO, USA). The mouse monoclonal anti myosin heavy chain (MAB4470) antibody was from R&D systems (Minneapolis, MN, USA); the WB secondary antibodies were horseradish peroxidase-conjugated goat anti-rabbit IgG and anti-mouse IgG (Sigma-Aldrich, St. Louis, MO, USA), while the fluorescent antibody was Alexa Fluor 488-conjugated goat anti-rabbit IgG (Invitrogen, Carlsbad, CA, USA).

### 4.3. Western Blot Analysis

The proteins were resolved by SDS-PAGE, blotted onto a nitrocellulose membrane, and probed with selected antibodies. The secondary antibodies were horseradish peroxidase-conjugated, and blots were developed with the Clarity ECL chemiluminescent substrate from BioRad (Hercules, CA, USA) or Trident Femto chemiluminescent substrate from Genetex (Irvine, CA, USA). Chemiluminescent signals were digitally acquired with Alliance Mini HD9 Imaging System (Uvitec, Cambridge, UK). Densitometry was performed with the ImageJ software (version 1.53t). The intensities of sarcoglycan bands were normalized for the intensity of the total protein loading, evaluated by Ponceau Red staining of the membranes, GAPDH, or β-actin.

### 4.4. Biotinylation of Surface Proteins

The biotinylation reaction was conducted according to [22]. At the end of the experiments, e.g., after 72 h of treatment with the C17 molecule, the myotubes were incubated at 4 °C for at least 10 min and all the procedures were performed at this temperature to slow down all cellular processes and, in particular, cell membrane protein recycling. The cells were then washed three times with ice cold PBS supplemented with calcium and magnesium and incubated under gentle agitation with a solution of 0.5 mg/mL of biotin (EZ-Link Sulfo-NHS-LC-Biotin, Thermo Fisher Scientific, Waltham, MA, USA) in PBS for 20 min at 4 °C. The biotinylation reaction was stopped before washing the cells twice with 100 mM of glycine in PBS (each wash was 5 min), and twice with PBS. The cells were lysed with RIPA buffer, and the lysates were centrifuged at 15,000× *g* at 4 °C for 30 min. The supernatants were quantified by BCA assay, and 50 µg of the proteins were incubated with streptavidin agarose beads (Thermo Fisher Scientific, Waltham, MA, USA) (20 µL of resin for each sample) over night at 4 °C under gentle rotation. The streptavidin resin was washed three times with RIPA buffer, and the bound proteins were eluted with Laemmli sample buffer for the western blot analysis. As a negative control, the lysate of non-biotinylated cells was incubated with the streptavidin resin and analyzed through Western blot analtsis to exclude nonspecific binding to the streptavidin resin. The absence of biotin internalization was assessed by probing the Western blot membranes with an antibody specific for the cytosolic protein β-actin.

### 4.5. Confocal Immunofluorescence

Immunofluorescence-confocal analyses were performed both in intact (not permeabilized) and permeabilized cells. Cells were grown on gelatin 0.02%-coated glasses. At the end of the experiments, non-permeabilized cells were incubated for 40 min at 4 °C to slow down protein membrane recycling, then gently washed twice with ice-cold PBS and incubated with primary antibodies for 5 h at 4 °C. After three gentle washings with ice-cold PBS, the cells were incubated with fluorescently labeled secondary antibodies for 1.5 h at 4 °C. Primary and secondary antibodies were diluted in PBS supplemented with 0.5% BSA. After secondary antibody incubation, cells were washed with PBS and then fixed for 15 min with 4% paraformaldehyde in PBS (PFA). After incubation with 50 mmol/L of NH_4_Cl for 15 min and washing with PBS, the nuclei were stained with DAPI. For immunofluorescence analysis of permeabilized cells, they were at first fixed with 4% PFA, washed with 50 mmol/L of NH_4_Cl, and then permeabilized with 0.5% Triton X-100. The permeabilized cells were then probed with the selected antibodies as above describe, with the omission of the last step of fixing. The cells were examined with a TCS-SP5 (Leica Mycrosystems, Wetzlar, Germany) or with a LSM 800 (Carl Zeiss, Jena, Germany) confocal laser scanning microscopes, as indicated.

### 4.6. Hypo-Osmotic Stress and CK Release Assay

A CK release assay was conducted according to [37]. Briefly, the myotubes differentiated for 7 days and treated for the last 72 h with 1‰ DMSO or C17 10 µM were incubated with two hypo-osmotic solutions (260 and 200 mOsm) for 20 min at 37 °C. The hypo-osmotic solutions consisted of a salt solution (5 mM of HEPES, 5 mM of KCl, 1 mM of MgCl_2_, 5 mM of NaCl, 1.2 mM of CaCl_2_, and 1 mM of glucose) supplemented with 157 mM of sucrose (200 mOsm) or 213 mM of sucrose (260 mOsm). Osmolarities were verified with an OM 801 osmometer (Vogel). After the treatment, the supernatant containing the released CK was removed and an equal volume of ice-cold hypo-osmotic solution was added to the cells. The cells were recovered by scraping and were lysed with T-PER^TM^ buffer (Thermo Fisher Scientific, Waltham, MA, USA). The released and intra-cellular creatine kinase were measured in sextuplicate using a Creatine Kinase Activity Colorimetric Assay Kit (BioVision) according to the manufacturer’s instructions.

### 4.7. Statistical Analysis

Values are expressed as means ± standard deviation (SD), as indicated. Statistical differences among the two groups were determined using Prism, Version 10.1.2 GraphPad software (San Diego, CA, USA) by a non-parametric Mann–Whitney test. If more than two groups were compared, the statistical differences were determined by a one-way ANOVA test, followed by an appropriate multiple comparisons test, as indicated. A level of confidence of *p* ≤ 0.05 was used for statistical significance.

## Data Availability

The data presented in this study are available in the article and in the Supplementary Materials.

## Supplementary Materials

The following supporting information can be downloaded at: https://www.mdpi.com/article/10.3390/ijms252413313/s1.
