# Supplementary material for: Efficacy of Cystic Fibrosis Transmembrane Regulator Corrector C17 in Beta-Sarcoglycanopathy—Assessment of Patient’s Primary Myotubes
Source: Int J Mol Sci. 2024 Dec 11;25(24):13313. doi: 10.3390/ijms252413313 (PMC11676211; doi:10.3390/ijms252413313)
Supplement: Supplementary file 1 [file ijms-25-13313-s001.zip › ijms-3302586-supplementary.pdf]

Supplementary Figure S1

A

```
# Length: 317
# Identity:     302/317 (95.3%)
# Similarity:   309/317 (97.5%)
# Gaps:           0/317 ( 0.0%)
# Score: 1571.0
#=======================================

hSGCB              2 AAAAAAAAEQQSSNGPVKKSMREKAVERRSVNKEHNSNFKAGYIPIDEDR     51
                       |||||||.|||.||||||||||||||||||:|||||||||||||||||||
mousesgcb          4 AAAAAAATEQQGSNGPVKKSMREKAVERRNVNKEHNSNFKAGYIPIDEDR     53

hSGCB             52 LHKTGLRGRKGNLAICVIILLFILAVINLIITLVIWAVIRIGPNGCDSME    101
                       ||||||||||||||||||:||||||||||:||||||||||||||||||||
mousesgcb         54 LHKTGLRGRKGNLAICVIVLLFILAVINLLITLVIWAVIRIGPNGCDSME    103

hSGCB            102 FHESGLLRFKQVSDMGVIHPLYKSTVGGRRNENLVITGNNQPIVFQQGTT    151
                     ||||||||||||||||||||||||||||||||||||||||||||||||||
mousesgcb        104 FHESGLLRFKQVSDMGVIHPLYKSTVGGRRNENLVITGNNQPIVFQQGTT    153

hSGCB            152 KLSVENNKTSITSDIGMQFFDPRTQNILFSTDYETHEFHLPSGVKSLNVQ    201
                     |||||.||||||||||||||||||.|||||||||||||||||||||||||
mousesgcb        154 KLSVEKNKTSITSDIGMQFFDPRTHNILFSTDYETHEFHLPSGVKSLNVQ    203

hSGCB            202 KASTERITSNATSDLNIKVDGRAIVRGNEGVFIMGKTIEFHMGGNMELKA    251
                     ||||||||||||||||||||||||||||||||||||||||||||::||||
mousesgcb        204 KASTERITSNATSDLNIKVDGRAIVRGNEGVFIMGKTIEFHMGGDVELKA    253

hSGCB            252 ENSIILNGSVMVSTTRLPSSSSGDQLGSGDWVRYKLCMCADGTLFKVQVT    301
                     ||||||||:||||.|||||||||||.||||||||||||||||||||||||
mousesgcb        254 ENSIILNGTVMVSPTRLPSSSSGDQSGSGDWVRYKLCMCADGTLFKVQVT    303

hSGCB            302 SQNMGCQISDNPCGNTH    318
                     ..|||||:|||||||||
mousesgcb        304 GHNMGCQVSDNPCGNTH    320
```

B

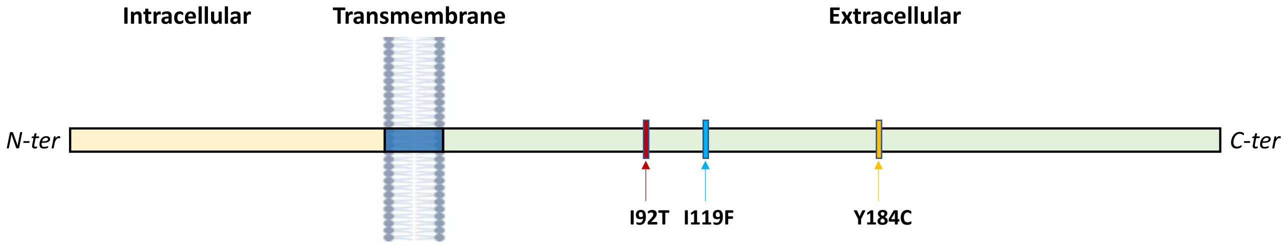

Supplementary Figure S1: β-sarcoglycan protein sequence.

(A) Sequence alignment of human and mouse β-sarcoglycan proteins is reported. Aminoacidic identity is indicated by a line, similarity by a dot. Highlighted residues correspond to the sites were mutations have been introduced into the mouse sequence.

(B) Schematic representation of β-sarcoglycan protein topology. Mutations analyzed in this study are highlighted. Modified from *Biorender.com.*

Supplementary Figure S2

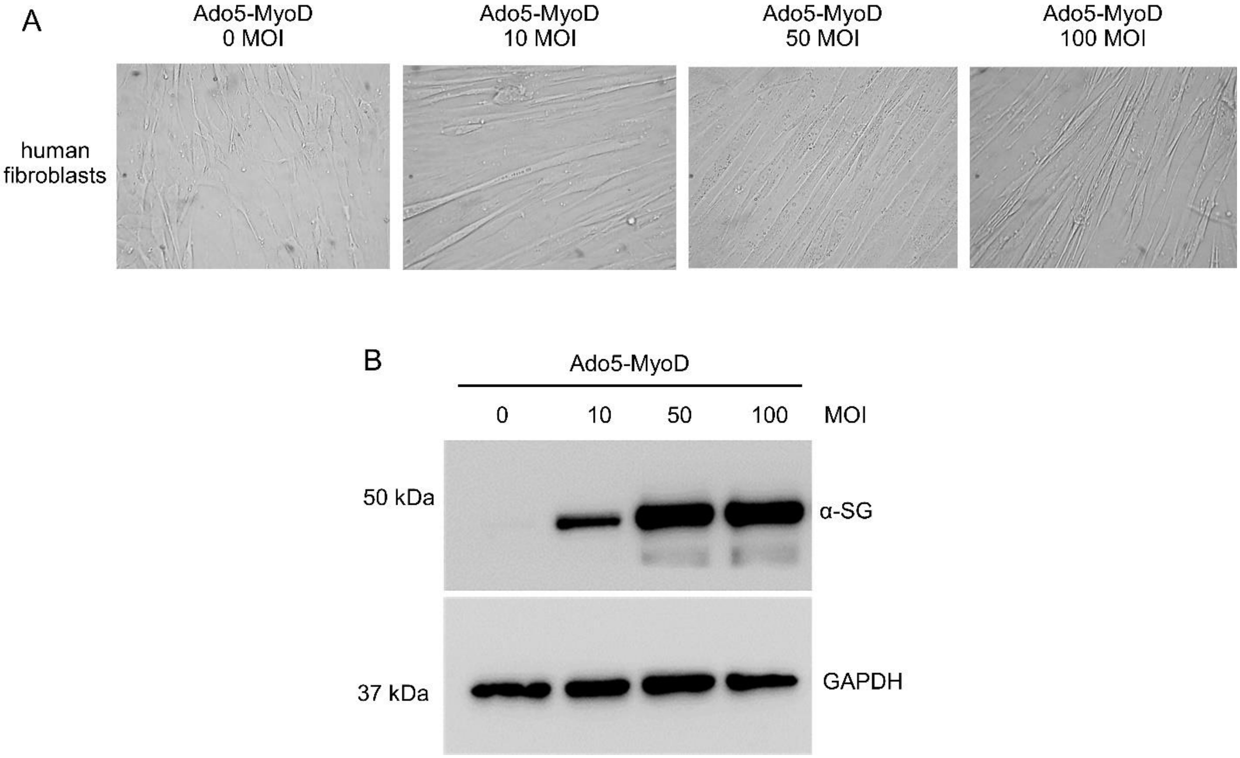

Supplementary Figure S2. **Transduction of human fibroblasts with the MyoD-expressing adenovirus (Ado5-MyoD) resulted in well differentiated myotubes.**

(A) phase contrast images of human fibroblasts untransduced (0 MOI) or transduced with the virus at the indicated MOI. Cells, after transduction, were maintained for 7 days in differentiation medium.

(B) Representative western blot of total protein lysates from cells at the end of the differentiation time; membranes were probed with antibodies specific to α-SG and GAPDH, used as loading control.

Ado5-MyoD = $1.5*10^{10}$ PFU/ml

MOI 10 = $7*10^{6}$ PFU

MOI 50 = $35*10^{6}$ PFU

MOI 100 = $70*10^{6}$ PFU

PFU: plaque-forming unit

MOI: multiplicity of infection

Supplementary Figure S3

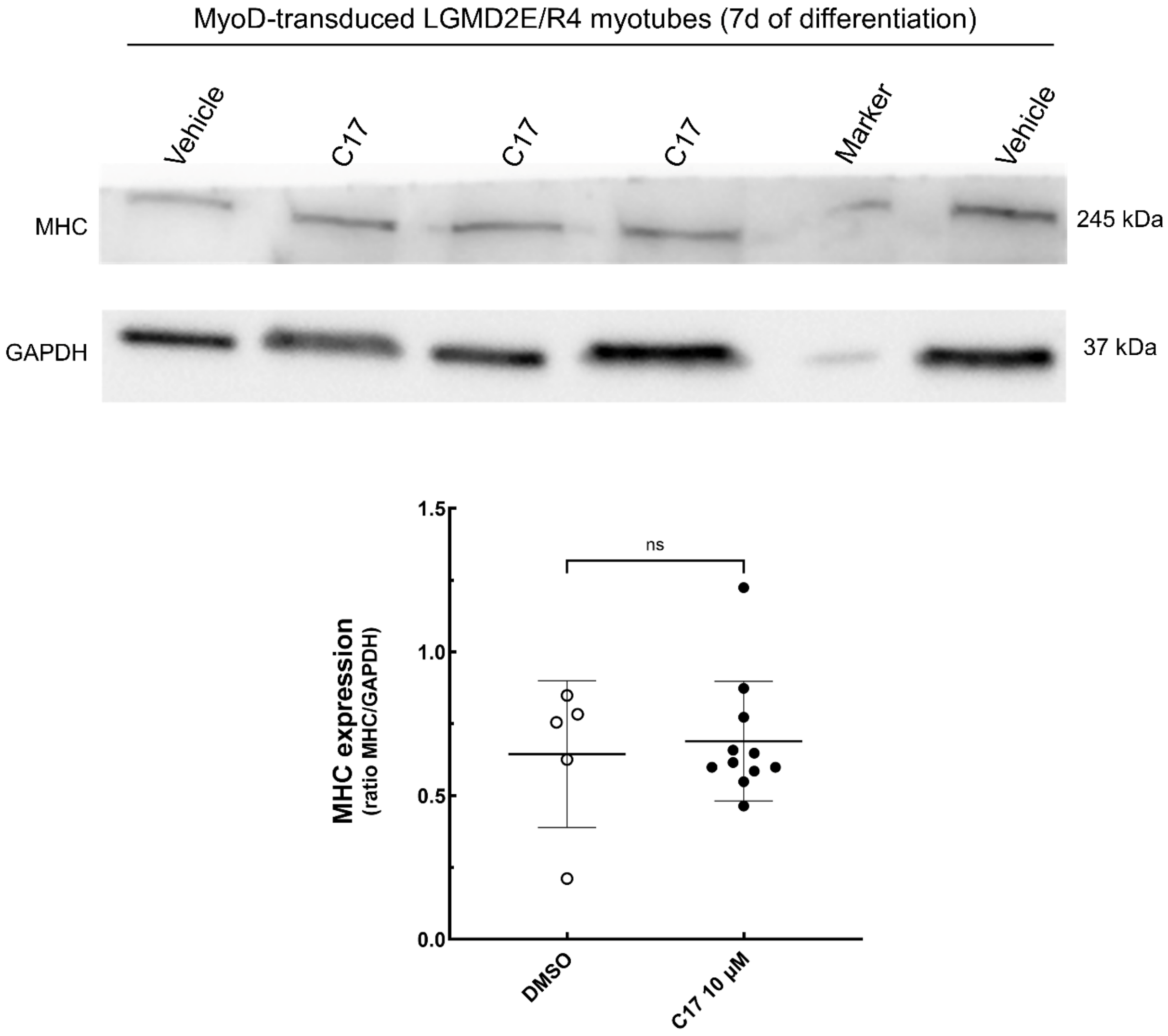

Supplementary Figure S3. Extent of differentiation of MyoD-transduced LGMD2E/R4 myotubes.

Representative western blot of total protein lysates from cells at the end of the differentiation time (7 days) treated for the last 96 hours with vehicle 1 ‰ (DMSO) or C17 10 µM; membranes were probed with antibodies specific to myosin heavy chain (MHC) and GAPDH, used as loading control. Below the western blots the densitometric analysis is reported, showing that there is no difference in differentiation among different samples. The mean value ± SD is also reported. Statistical analysis was performed by the Mann-Whitney test.
